# Cocktails of probiotics pre-adapted to multiple stress factors are more robust under simulated gastrointestinal conditions than their parental counterparts and exhibit enhanced antagonistic capabilities against *Escherichia coli* and *Staphylococcus aureus*

**DOI:** 10.1186/s13099-015-0053-5

**Published:** 2015-02-26

**Authors:** Moloko Gloria Mathipa, Mapitsi Silvester Thantsha

**Affiliations:** Department of Microbiology and Plant Pathology, New Agricultural Sciences Building, University of Pretoria, Pretoria, 0002 South Africa

**Keywords:** Antipathogenic, *Bifidobacterium*, Gastrointestinal, *Lactobacillus*, Multi- stress, Probiotics

## Abstract

**Background:**

The success of the probiotics in delivery of health benefits depends on their ability to withstand the technological and gastrointestinal conditions; hence development of robust cultures is critical to the probiotic industry. Combinations of probiotic cultures have proven to be more effective than the use of single cultures for treatment and prevention of heterogeneous diseases. We investigated the effect of pre- adaptation of probiotics to multiple stresses on their stability under simulated gastrointestinal conditions and the effect of their singular as well as their synergistic antagonistic effect against selected enteric pathogens.

**Methods:**

Probiotic cultures were inoculated into MRS broth adjusted to pH 2 and incubated for 2 h at 37°C. Survivors of pH 2 were subcultured into 2% bile acid for 1 h at 37°C. Cells that showed growth after exposure to 2% bile acid for 1 h were finally inoculated in fresh MRS broth and incubated at 55°C for 2 h. The cells surviving were then used as stress adapted cultures. The adapted cultures were exposed to simulated gastrointestinal conditions and their non- adapted counterparts were used to compare the effects of stress adaptation. The combination cultures were tested for their antipathogenic effects on *Escherichia coli* and *Staphylococcus aureus.*

**Results:**

Acid and bile tolerances of most of the stress-adapted cells were higher than of the non-adapted cells. Viable counts of all the stress-adapted lactobacilli and *Bifidobacterium longum* LMG 13197 were higher after sequential exposure to simulated gastric and intestinal fluids. However, for *B. longum* Bb46 and *B. bifidum* LMG 13197, viability of non-adapted cells was higher than for adapted cells after exposure to these fluids. A cocktail containing *L. plantarum* + *B. longum* Bb46 + *B. longum* LMG 13197 best inhibited *S. aureus* while *E. coli* was best inhibited by a combination containing *L. acidophilus* La14 150B + *B. longum* Bb46 + *B. bifidum* LMG 11041. A cocktail containing the six non- adapted cultures was the least effective in inhibiting the pathogens.

**Conclusion:**

Multi-stress pre-adaptation enhances viability of probiotics under simulated gastrointestinal conditions; and formulations containing a mixture of multi stress-adapted cells exhibits enhanced synergistic effects against foodborne pathogens.

## Background

The human gastrointestinal tract (GIT) is a home to a community of microorganisms, present in great richness and complexity [1,2]. There are different bacteria, both beneficial and harmful, present throughout the GIT, in the different niches from the mouth to the colon. Health effects associated with the beneficial microflora have led to the development of probiotics products. Probiotics are defined as ‘live microorganisms which when administered in adequate amounts, confer a health benefit on the host’ [3]. They play a role in the stabilisation of the intestinal microflora by competition against pathogens [4], reduction of lactose intolerance [5], prevention of antibiotic-induced diarrhoea [6] and stimulation of the immune system [7], just to name a few. In order for a microorganism to be referred to as a probiotic; among other criteria, it must exhibit resistance to technological processes used in preparing the vehicle of probiotic delivery and produce antimicrobial substances [3,8,9].

Probiotics are taken in the form of functional foods such as fermented milk and cheese and also as pharmaceutical preparation e.g. capsules. They are used as starter cultures and they therefore undergo all the stress factors during production and storage. After their storage, they are consumed and pass through the GIT where they are exposed to conditions such as low pH and high bile concentrations. These technological and gastrointestinal factors present a significant challenge to the probiotic industry. In order for probiotic cells to confer their beneficial effects to the host, they have to survive in high numbers [10]. Many probiotic bacteria have shown to die in the food products after exposure to low pH during fermentation, oxygen during refrigeration, distribution and storage of products, and/or acid in the human stomach [11]. The adaptation of the probiotic strains to different challenges that they are faced with during their production and administration is therefore crucial for their survival. Previous researchers have reported that the pre- exposure of the probiotic cultures to stressful conditions enhances their stability when subsequently exposed to those stressful conditions [12,13].

The use of single bacterial cultures has been studied since the discovery of probiotics and the need to enhance their effects led to introduction of the use of probiotic combinations. Previous studies for the effectiveness of probiotic strains, reported that multi-strain probiotics showed greater efficacy than the single strain preparations [14]. A mixture of *B. bifidum* BGN4, *B. lactis* AD011 and *L. acidophilus* AD031 was an effective approach in preventing the development of eczema in infants at high risk of allergy during the first year of life than single probiotic cultures [15]. There are number of products available on the market that contain combinations of probiotic cultures. VSL #3 combines eight different probiotic bacteria, has been used in different studies and shown to have better effects than the single strain [16]. Other probiotic mixtures, Ecological® Relief (*Bifidobacterium bifidum* W23*, Bifidobacterium lactis* W52*, Bifidobacterium longum W108, Lactobacillus casei W79, Lactobacillus plantarum* W62 *and Lactobacillus rhamnosus*W71) and PrimaLac (*Lactobacillus acidophilus, Lactobacillus casei, Enterococcus faecium* and *Bifidobacterium bifidum*) are among the multi- species probiotics that have been shown to perform better than the single strain probiotics [17,18], just to name a few. However, there is limited knowledge on the effects of pre- adaptation of probiotic cells to more than one stress factor before they are used for multi- strain preparations. Taking these into consideration, the current study aimed to enhance the stability of probiotics under simulated gastrointestinal conditions through pre-adaptation to acid- bile- temperature. This is done by looking at the effects of stress adaptation through the exposure of the cells to the gastric and the intestinal conditions. We also going to determine the antipathogenic effects of the different multi- stress adapted probiotic combinations on *E. coli and S. aureus*.

## Results and discussion

### Acid- bile- temperature adaptation

The use of probiotics is increasing at a very fast rate as their importance is seen throughout the world, however, their sensitivity hinders them from being used. The definition of probiotics highlights the importance of maintaining high viable number of microorganisms throughout the entire shelf-life of the products into which they are incorporated. These products must contain a number of viable cells shown to be efficacious, which is generally 10^6^ – 10^8^ cfu/ ml or g [19]. However, a number of reports indicate that there is a relatively poor survival of probiotic strains during most of the technological processes used by the food industry [20,21] and therefore most products do not contain the required number of viable microorganisms. Poor viability of probiotics stimulated research interest into different methods to protect or improve their viability. The use of different strategies on probiotics strains to enhance their stability, viability and functionality has been studied and reviewed in most recent probiotics work [22,23]. The optimization of strategies based on stress adaptation and cross protection mechanisms therefore constitute an attractive option to improve performance and functionality of probiotics [24]. It has been shown earlier that the exposure of probiotics to sub- lethal stress for the enhancement of stress responses has been found to be highly effective [25]. Taking those studies into consideration, the current study investigated the effect of successive pre-adaptation of probiotic strains to multiple stress factors corresponding to those they encounter during processing and after ingestion, specifically acid, bile and high temperature, on their (probiotics) stability when later exposed to those similar individual factors.

**Figure 1:**
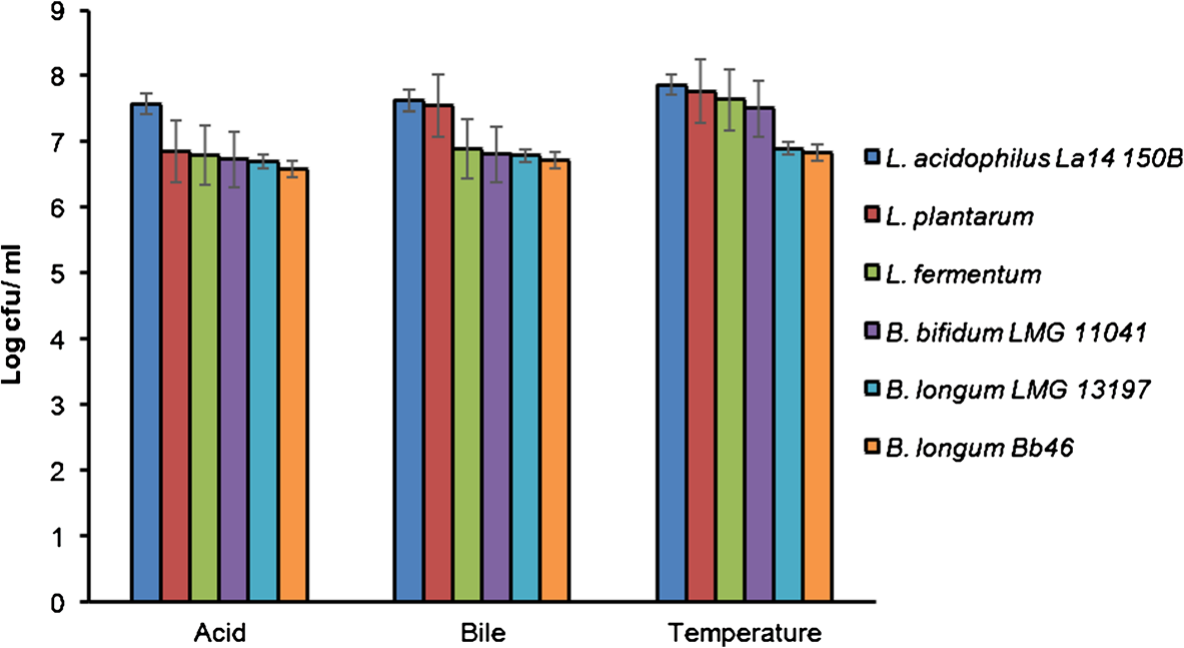
**Viable counts of probiotics after their exposure to acid, bile and temperature.** *The log cfu/ ml of the probiotic cultures were analysed and calculated at the end of each stress adaptation step. Each bar represents the mean of three independent experiments, error bars are standard deviations.*

Performance of the six commercial strains of probiotics, *Lactobacilli* and *Bifidobacteria* during pre- adaptation to acid- bile- temperature is shown in Figure 1. The standard pH for the acidity that the bacteria have to be able to survive in is pH 2 [26]. Taking that into consideration, we exposed the six commercial strains to pH 2 for 120 minutes for their pre- exposure to acid. The number of surviving cells for both the *Lactobacilli* and the *Bifidobacteria* cells ranged from 6.58 to 7.57 log cfu/ml, with the *Lactobacilli* cells more tolerant to acid than *Bifidobacteria* cells. The best surviving cells *L. acidophilus* La14 150B had final log cfu/ml of 7.57, meaning that through the exposure to acid 0.43 log cfu/ ml cells did not survive. Viable cell reductions of 1.15, 1.2, 1.27, 1.30 and 1.4 log cfu/ml were recorded for *L. plantarum*, *L. fermentum*, *B. bifidum* LMG 11041, *B. longum* LMG 13197 and *B. longum* Bb46, respectively. The surviving cells were taken as the acid adapted and then they were subcultured for use in bile adaptation study.

Not only do probiotics have to be able to survive in low acid environment, they also have to be able to grow in high bile concentration for them to confer health effects on the host. We, therefore, exposed the acid adapted cells to bile salts to check for their survival in the intestinal conditions. The bile salt concentration of 2% was used as the standard here in our study as it represents most extreme concentration that can be found in the human intestine during the first hour of digestion [27,28]. The number of the cells that survived at 2% bile salt concentration for 120 minutes were calculated for each bacterial culture. From the initial concentration (10^8^ log cfu/ ml) there was a decrease in the number of surviving cells in all the probiotic cultures. The log cfu/ ml of the cultures ranged from 6.72 to 7.62 for all the cultures (Figure 1). There was a decrease of 0.38, 0.46, 1.11, 1.19, 1.22 and 1.29 log units for *L. acidophilus* La14 150B, *L. plantarum*, *L. fermentum*, *B. bifidum* LMG 11041, *B. longum* LMG 13197 and *B. longum* Bb46, respectively. It was interesting and worth noting that even though there was a decrease in the viable numbers of the strains during exposure to bile salts for the cultures, reduction for the strains was lower compared to when the original strains were exposed to acid. The observed less reduction in viability is attributed to pre-exposure to acid, which increased stability of strains. Since 2% bile salt concentration is the extreme condition, cells that survived after their exposure were regarded as acid- bile adapted cells and were then subsequently used in high temperature adaptation experiments.

We then lastly incorporated high temperature into the stress adaptation process. The acid- bile adapted cells were then incubated at 55°C for 120 mins. The surviving cells ranged from 6.82 to 7.86 log cfu/ ml for all the probiotic cells. There was a decrease in the log cfu/ml with a difference of 0.14, 0.24, 0.37, 0.49, 1.11 and 1.17 log cfu/ ml for *L. acidophilus* La14 150B, *L. plantarum*, *L. fermentum*, *B. bifidum* LMG 11041, *B. longum* LMG 13197 and *B. longum* Bb46, respectively in order from the best to the least surviving strain (Figure 1). The order from the best to the least surviving strain was the same as was observed for acid and bile adaptation experiments. The *Lactobacilli* strains survived better that the *Bifidobacteria* strains throughout the whole stress adaptation process, indicating that *Lactobacilli* strains are more resistant than *Bifidobacteria* strains. This is in agreement with an earlier statement by Sanz [24] that *Bifidobacterium* strains are highly sensitive strains when compared to *Lactobacillus* strains. Similar to what was observed during acid and bile adaptation studies, reduction in viable numbers after exposure to 55°C was the lowest for acid-bile adapted cells compared to parental cells during acid adaptation, and acid-adapted cells during bile adaptation.

Previous study to investigate the effect of different single stress factors on the survival of probiotics in the GIT tract concluded that the stress adaptation to either acid or bile did not show statistical relevant positive effect [29]. The results found in [30] showed that when they pre- treated probiotics to temperature (50°C, 30 mins) the viability reduction trend was the same as with the non- treated cells. However, when these researchers further conducted a study using combined stress factors, they reported that *Bifidobacterium* isolates pre- treated with acid- bile- NaCl showed improved properties when they were later exposed to acid, bile and NaCl conditions, indicating that pre- exposure to combined stress factors had better effects than when using single stress adapted cells. This suggested that multi- stress pre- treatment may be useful to enhance the stability and the functional properties of the probiotics [31]. It is for this reason that, in our current study, we further pre- treated the acid- adapted cells to high bile and temperature, to make the cells more robust when exposed to stress later on. We envisaged that initial stress adaptation process will enhance survival of the probiotics when further exposed to single stress factors. Our results showed a step by step improvement of the survival of the probiotics when they were pre- treated with acid, bile and then temperature. These results therefore demonstrate that pre- treatment of probiotic cells to acid- bile- temperature made them more stable than the acid adapted and acid- bile adapted cells. The acid-bile-temperature adapted cells were significantly more stable than both the acid adapted cells (p = 0.041) and acid-bile adapted cells (p = 0.036). This is confirmation that the multiple stress pre- adapted cells are better cells to use when compared to the single stress adapted cells and the non- adapted cells.

### Survival of the non- adapted and acid- bile- temperature adapted probiotic strains in acid and bile

Oral probiotic strains experience severe acidic conditions in the stomach, where the pH is close to 2 [1]. After the cells pass through the acidic stomach, they are exposed to bile salts in the intestine, where the normal concentration is around 0.3%, but can range up to the extreme of 2.0% [27]. Both these factors strongly compromises bacterial viability. Resistance of these strains to acid and bile upon ingestion is therefore crucial in the production of probiotic products [32]. We studied the survival of the acid- bile- temperature adapted cells in the presence of the different acidic and bile concentration comparing them to their respective non- adapted cells. The initial concentrations of both the non- adapted and adapted cells were adjusted to 10^8^ cfu/ ml.

### Acid resistance

Table 1 shows the survival of the non- adapted and the adapted cells in pH 2, 2.5 and 3 over a period of 180 mins. From the results, the survival of the cells ranged from 6.43 to 7.98 log cfu/ ml, with the adapted cells surviving better than the non- adapted cells. The order from the best to the least acid tolerant strains, for both adapted and non- adapted cells was *L. acidophilus* La14 150B > *L. plantarum* > *L. fermentum > Bifidobacterium bifidum* LMG 11041 > *B. longum* LMG 13197 > *B. longum* Bb46. Survival of all the acid- bile- temperature adapted cells in acid was significantly higher than the non- adapted, with p- values of 0.0257, 0.0448, 0.0464, 0.0018, 0.0452 and 0.0431 for *Bifidobacterium bifidum* LMG 11041, *B. longum* LMG 13197 *B. longum* Bb46, *L. fermentum, L. plantarum* and *L. acidophilus* La14 150B, respectively. Our recorded higher counts in the higher counts for stress adapted strains than the non- adapted cells confirm that pre- adaptation to stress does provide protection to the cells enhancing their growth.

**Table 1:** **The counts showing acid tolerance of the non- adapted and the adapted cells of the probiotics over time**

**Probiotic strains**	**Acid- bile- temperature adapted**	**pH**								
		**2**			**2.5**			**3**		
		**Time (mins)**								
		**60**	**120**	**180**	**60**	**120**	**180**	**60**	**120**	**180**
	**Bacterial counts (Log cfu/ml)**									
*B. bifidum* LMG 11041	No	6.76 ± 1.1	6.63 ± 1.2	6.48 ± 1.1	6.71 ± 2.1	6.65 ± 1.2	6.54 ± 0.6	6.99 ± 3.2	6.93 ± 2.8	6.69 ± 1.5
	Yes	6.95 ± 1.5	6.84 ± 1.0	6.79 ± 1.2	6.88 ± 2.3	6.78 ± 2.5	6.72 ± 1.7	6.98 ± 2.8	6.95 ± 2.3	6.88 ± 2.0
*B. longum* LMG 13197	No	6.76 ± 1.2	6.67 ± 1.5	6.53 ± 1.0	6.66 ± 1.2	6.64 ± 0.8	6.58 ± 1.6	6.86 ± 1.7	6.81 ± 2.0	6.74 ± 2.2
	Yes	6.87 ± 2.5	6.82 ± 1.2	6.75 ± 1.5	6.89 ± 2.5	6.79 ± 1.5	6.64 ± 2.0	6.93 ± 3.5	6.87 ± 2.7	6.79 ± 2.5
*B. longum* Bb46	No	6.62 ± 1.5	6.57 ± 1.1	6.43 ± 1.2	6.69 ± 0.5	6.62 ± 1.1	6.54 ± 1.8	6.65 ± 2.0	6.6 ± 2.3	6.54 ± 1.7
	Yes	6.82 ± 2.0	6.77 ± 2.3	6.64 ± 1.8	6.79 ± 1.0	6.65 ± 1.2	6.58 ± 1.5	6.63 ± 2.5	6.6 ± 2.1	6.57 ± 2.1
*L. acidophilus* La14 150B	No	7.88 ± 1.2	7.76 ± 0.8	7.69 ± 1.2	7.79 ± 2.2	7.67 ± 2.5	7.56 ± 2.8	7.97 ± 2.2	7.79 ± 2.0	7.68 ± 2.1
	Yes	7.97 ± 1.5	7.91 ± 0.3	7.81 ± 1.2	7.94 ± 3.3	7.87 ± 2.8	7.72 ± 2.0	7.98 ± 2.0	7.83 ± 1.8	7.79 ± 2.3
*L. fermentum*	No	7.72 ± 0.7	7.57 ± 1.3	7.52 ± 0.5	7.88 ± 1.5	7.68 ± 1.8	7.51 ± 2.0	7.81 ± 1.2	7.59 ± 1.5	7.54 ± 1.8
	Yes	7.87 ± 1.5	7.89 ± 0.8	7.79 ± 1.2	7.85 ± 2.5	7.83 ± 2.1	7.76 ± 1.8	7.89 ± 2.6	7.8 ± 1.8	7.77 ± 1.5
*L. plantarum*	No	7.93 ± 1.3	7.72 ± 1.5	7.61 ± 1.2	7.97 ± 1.9	7.79 ± 0.9	7.69 ± 0.2	7.93 ± 1.8	7.86 ± 1.2	7.68 ± 1.5
	Yes	7.94 ± 2.3	7.85 ± 1.7	7.78 ± 1.3	7.99 ± 2.0	7.89 ± 1.6	7.79 ± 1.2	7.98 ± 2.5	7.93 ± 2.2	7.86 ± 2.3

Each value in the table represents the mean of triplicate plate count readings from three separate experiments. The table shows the trend that was followed by the adapted and the non- adapted cells when they were exposed to the different pH values.

Researchers elsewhere reported the effect of pre- adaptation of various probiotic to different stress factors to enhance their growth when they are further exposed to the stress factors. Previous study by [22] reported that pre- adaptation of *L. acidophilus* to acid stress (pH 5.0, 60 min) was found to confer the resistance against subsequent exposure to pH 3. Lorca and de Valdez [25] reported that *L. acidophilus* pre-exposed to acid (pH 3, 60 mins) survived better than the non- acid treated cells. Similarly, Park et al. [33], reported that pre-adaptation of *B. breve* cells to pH 5.2 protected them against subsequent lethal pH values of 2.0–5.0. Our results are therefore in agreement with these previous studies. However, contrary to these studies in which the probiotics were pre-adapted to a single stress factor, in our study the probiotics were pre-adapted to multiple stress factors, namely, acid, − bile and high- temperature in order to further enhance growth of the cells. In a previous study by [34] they reported that *B. longum* is acid- sensitive and that its acid- adaptation would not enhance its acid tolerance enough. It was interesting to observe that in our study, after the pre- adaptation of *B. longum* cells to multiple stress factors, they managed to grow in the acidic environment. This suggests that pre-adaptation to multiple stress increases stability of even the sensitive strains better than single stress adaptation. The survival in high number of the adapted cells that were used here in our study indicate that the cells could survive in the acidic stomach therefore reaching the areas of beneficial activity [27] in adequate numbers which is in accordance with the criterion that cells must be able to survive in large numbers.

### Bile resistance

The ability to survive bile concentrations produced in the human small intestines and to take up residence and multiply in human large intestine is another important characteristic of probiotics [35]. Different researchers use different bile salt concentrations for bile tolerance studies, with a range of 0.5 to 2.0% (w/v) most preferred. In our study we therefore looked at the survival of the non-adapted and adapted cells in 0, 1.0, 2.0 and 3.0% bile salts concentration. Table 2 shows the survival of the non- adapted and the adapted cells in the different bile salt concentrations over time. There was an increase in the number of the surviving cells in 0% bile concentrations throughout the whole incubation. When the cells were exposed to 1.0, 2.0 and 3.0% bile salts concentrations, there was a decrease in the number of surviving cells where the higher the bile salt concentration, the lower the number surviving. The number of the surviving cells ranged from 5.74 to 9.68 log cfu/ ml, with the adapted cells surviving better than the non- adapted cells in all the bile salt concentrations. Tolerance of the multiple stress adapted cells to bile salts was significantly higher than of the non- adapted ones for all the tested strains at the end of incubation in all bile concentrations represented by the p- values: 0.043, 0.031, 0.0042, 0.029, 0.037 and 0.0039 for *B. bifidum* LMG 11041, *B. longum* LMG 13197, *B. longum* BB46, *L. acidophilus* La14 150B, *L. fermentum* and *L. plantarum*, respectively. In previous different studies the survival of the adapted cells being better than the non- adapted cells has been reported [36-38]. The percentage of survival of the bile- adapted *Bifidobacterium* strains was better than the corresponding parental cells when exposed to bile salts in a study by Kim et al. [37]. Another study by [38] reported that the difference between the *Bifidobacterium* parental and the bile- adapted strain showed statistically significant difference in favour of the adapted strains. Improved survival of stress adapted strains than their non-adapted counterparts under all concentrations of bile could be attributed to an increase in F_1_F_0_-ATPase activity produced by acquisition of bile resistance [38].

**Table 2:** **The viable counts of the non- adapted and the adapted cultures exposed to different bile concentrations**

**Probiotic strains**	**Acid- bile- temperature adapted**	**Bile concentration (%)**											
		**0**			**1**			**2**				**3**	
		**Time (hours)**											
		**1**	**2**	**3**	**1**	**2**	**3**	**1**	**2**	**3**	**1**	**2**	**3**
*B. bifidum* LMG 11041	No	8.07 ± 1.5	8.15 ± 2.2	8.28 ± 2.6	7.22 ± 1.5	7.07 ± 2.4	6.91 ± 1.8	6.95 ± 2.0	6.83 ± 1.2	6.71 ± 0.5	6.67 ± 2.6	6.38 ± 1.8	6.22 ± 1.4
	Yes	8.46 ± 1.7	8.72 ± 2.7	9.05 ± 2.5	7.65 ± 2.5	7.54 ± 2.1	7.43 ± 1.9	7.47 ± 2.0	7.31 ± 1.5	7.21 ± 1.0	7.34 ± 1.9	7.22 ± 2.3	7.12 ± 1.5
*B. longum* LMG 13197	No	8.05 ± 1.7	8.12 ± 2.0	8.23 ± 2.6	7.12 ± 0.9	6.92 ± 1.5	6.73 ± 1.2	6.80 ± 1.8	6.73 ± 1.0	6.51 ± 2.0	6.45 ± 2.0	6.28 ± 1.25	6.13 ± 1.3
	Yes	8.44 ± 2.3	8.61 ± 2.9	8.83 ± 3.1	7.51 ± 1.2	7.42 ± 2.0	7.37 ± 1.5	7.45 ± 2.5	7.38 ± 2.8	7.22 ± 2.0	7.27 ± 2.3	7.13 ± 2.5	7.05 ± 1.5
*B. longum* Bb46	No	8.03 ± 1.2	8.08 ± 2.0	8.19 ± 2.7	7.07 ± 2.0	6.84 ± 1.8	6.63 ± 1.0	6.71 ± 2.0	6.63 ± 1.5	6.47 ± 1.2	6.22 ± 1.1	6.04 ± 1.5	5.74 ± 0.9
	Yes	8.40 ± 2.5	8.55 ± 3.2	8.78 ± 3.7	7.47 ± 2.5	7.32 ± 1.8	7.27 ± 2.0	7.42 ± 2.5	7.32 ± 2.3	7.18 ± 2.0	7.07 ± 2.5	6.94 ± 2.0	6.76 ± 2.3
*L. acidophilus* La14 150B	No	8.31 ± 2.1	8.45 ± 2.5	8.54 ± 3.0	7.43 ± 1.5	7.22 ± 1.7	7.17 ± 2.0	7.23 ± 2.0	7.12 ± 1.5	7.05 ± 1.2	7.08 ± 1.8	6.94 ± 1.5	6.77 ± 2.0
	Yes	8.88 ± 3.4	9.37 ± 2.9	9.68 ± 2.4	7.92 ± 2.6	7.83 ± 2.0	7.76 ± 2.3	7.84 ± 2.2	7.74 ± 2.0	7.61 ± 1.8	7.64 ± 1.5	7.52 ± 1.0	7.44 ± 1.1
*L. fermentum*	No	8.12 ± 2.1	8.18 ± 2.0	8.34 ± 2.5	7.26 ± 0.5	7.11 ± 1.2	7.02 ± 0.9	7.04 ± 1.5	6.94 ± 2.0	6.84 ± 1.2	6.87 ± 1.2	6.73 ± 1.5	6.51 ± 2.0
	Yes	8.58 ± 2.0	8.86 ± 2.7	9.17 ± 1.7	7.77 ± 1.5	7.70 ± 2.0	7.62 ± 1.7	7.52 ± 2.3	7.44 ± 2.7	7.38 ± 1.3	7.41 ± 1.8	7.28 ± 2.0	7.19 ± 2.3
*L. plantarum*	No	8.18 ± 1.5	8.24 ± 2.5	8.42 ± 1.8	7.38 ± 1.2	7.17 ± 1.0	7.08 ± 1.5	7.13 ± 2.0	7.08 ± 1.2	6.97 ± 1.5	6.94 ± 1.1	6.84 ± 1.3	6.73 ± 1.5
	Yes	8.63 ± 2.2	9.08 ± 1.5	9.47 ± 2.4	7.85 ± 2.0	7.77 ± 2.3	7.68 ± 1.8	7.67 ± 2.5	7.51 ± 2.3	7.42 ± 2.0	7.49 ± 2.5	7.39 ± 2.7	7.28 ± 2.3

Each value in the table represents the mean of triplicate counts from three separate experiments.

Overall the *Lactobacilli* cells survived better than the *Bifidobacteria* cells in both occasions. The results from [39] showed that *Lactobacillus acidophilus* is more resistant when compared with *Bifidobacterium* spp. and in a study comparing two genera of probiotics they showed that *Bifidobacterium* strains were reported to be more susceptible to loss than the lactobacilli cells [40]. In a previous study by [41] they maintained *Lactobacillus* and *Bifidobacterium* strains at bile concentrations of 0–1.5% for 3 hours, and their results showed that survival varied among the strains depending on the bile concentrations and exposure times. Therefore, our results confirm these studies. It is worth noting that in our study we pre- adapted the cells to acid- bile- temperature, not only to one stress factor. When comparing the adapted and the non- adapted cells for their survival in the acid and bile tolerance results, the adapted cells survived well than the non- adapted. Therefore we proved our objective that the multi- stress pre- adaptation can be used a safe mechanism to enhance survival of the probiotic under unfavourable conditions.

### Survival of probiotics cells after sequential exposure to simulated gastric and the intestinal conditions

Probiotic bacteria must be able to survive the transport to the active site, therefore be able to survive passage through the acidic environment to the stomach [42]. Furthermore they need to be able to colonise and survive in the small intestine in order for them to be able to exert positive effects on the health and well-being of the host [43]. Thus, they need to satisfy a criterion entailing their ability to survive the GIT processes, in the stomach and the intestinal tract [44]. As the two stresses of stomach transit and small intestinal transit might interact and thereby affect the viability of the strains in a synergistic fashion, it is important to evaluate all components (enzymes, low pH, bile salts and food vehicle) in one system, rather than evaluating the effect of each component in separate experiments [45].

**Figure 2:**
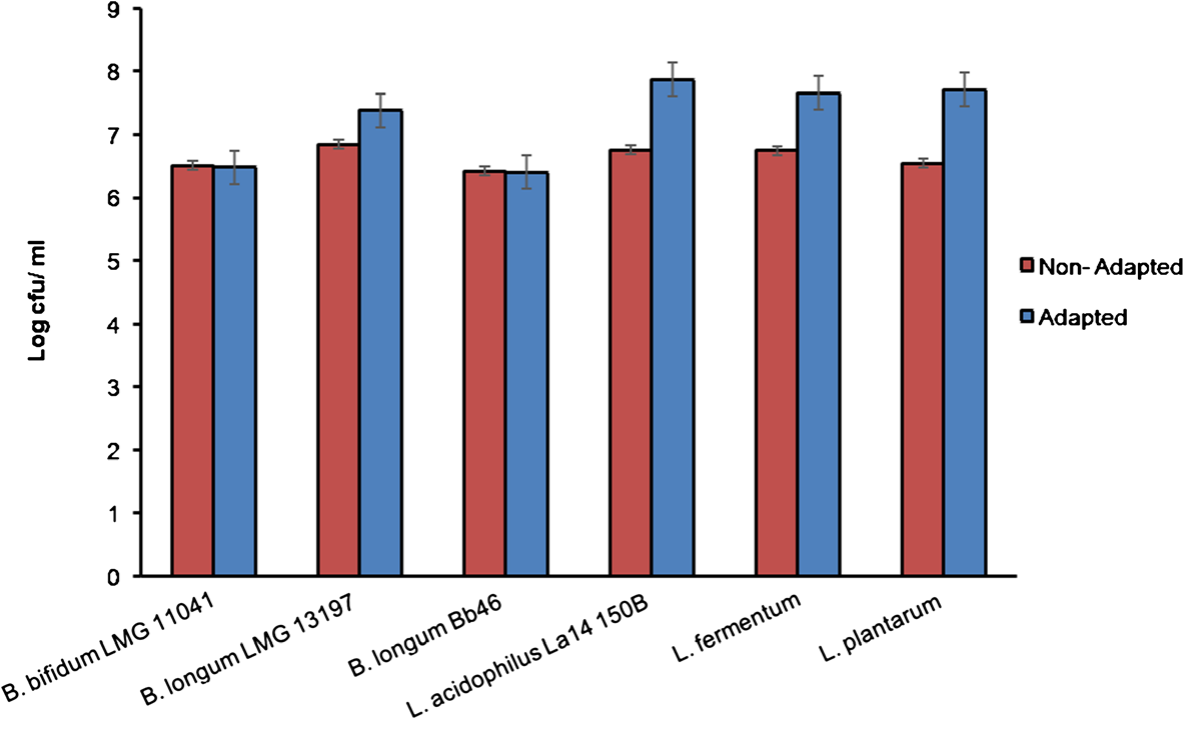
**Survival of non-adapted and multi-stress adapted probiotic strains during exposure to simulated gastric and intestinal conditions.** *Counts are the difference log cfu/ ml obtained after subtracting counts obtained after the exposure to gastric and intestinal conditions from the initial counts. Each point represents the mean of three independent experiments, error bars are standard deviations.*

We compared survival of the non- adapted cells with that of their acid- bile- temperature adapted counterparts after subsequent exposure to simulated gastric and intestinal fluids. Viable counts of all the multi-stress adapted *Lactobacilli* strains and *B. longum* LMG 13197cells were higher compared to that of the non- adapted cells (Figure 2). Adaptation improved survival of all *Lactobacilli* strains by ~1 log cfu/ml and of *B. longum* LMG13197 by 0.5 log cfu/ml. There was a significant difference between the non-adapted and adapted cells at the end of exposure period (p = 0.0002). On the contrary, for *B. longum* Bb 46 and *B. bifidum* LMG 11041, counts of non- adapted cells were higher than of the adapted cells (Figure 2). There was a difference of 1.11, 1.167, 0.911 and 0.534 log cfu/ ml between the adapted and non- adapted cells of *L. acidophilus* La14 150B, *L. plantarum*, *L. fermentum* and *B. longum* LMG 13179, respectively. The viable numbers of the non-adapted cells of *B. bifidum* LMG 11041 and *B. longum* Bb46 were higher than of their non-adapted counterparts by 0.026 and 0.014 log cfu/ ml, respectively. These results indicated that pre-exposure to multiple stresses did not improve stability of these two strains in simulated gastrointestinal fluid. Furthermore, it is worth noting that although survival was not improved for these strains, their stability in the simulated gastrointestinal fluids was not negatively affected by pre-exposure as the difference in viability between the non-adapted and adapted cells for the respective strains was negligible (non-significant).

In a different study to test for survival of the probiotic cells in the gastric and intestinal conditions, Pochart et al. [46], reported that the survival of cells of *L. acidophilus* and *B. bifidum* through the gastric and the intestinal conditions was not significantly different. We used the acid- bile- temperature adapted cells and checked for their survival in the simulated gastric and intestinal conditions. There was a significant (p = 0.0002) increase in the survival of the adapted cells when compared to the non- adapted cells. From our results we can therefore accept the hypothesis of this study that the pre- adaptation of the probiotic cells to acid- bile- temperature enhanced the growth of the probiotics in the sequential exposure to the simulated gastric and intestinal conditions. Previous literature by Drouault et al. [47], and Berrada et al. [48], has reported that *Lactobacillus acidophilus* and *Bifidobacteria* have been reported to be more resistant to gastric and intestinal conditions but large differences exist between strains. This was in accordance with our results as we saw both the non- adapted and the adapted cells surviving after the sequential exposure to the adapted and the non- adapted cells. In the previous study by Huang and Adams [44] they tested the survival of the cells in the simulated gastric and intestinal conditions separately. They reported that when the strains were first exposed to the gastric conditions, all strains showed progressive reduction in survival while the exposure to simulated intestinal conditions resulted in all strains retaining the same viability. The ability of the cells to survive the gastric and intestinal conditions means that the cells can be used as probiotics, since their survival suggests that they can be delivered to the intestine in high numbers [42]. The pre- adaptation of the probiotic strains to acid- bile- temperature therefore makes the adapted strains more desirable for the use as probiotic products.

### Antagonistic effects of single and probiotic cocktails on *S. aureus*

Antibiotics have always been the drugs of choice for the treatment of pathogens, but their ineffectiveness against some pathogens [49], as well as the problem of antibiotic resistance led to preference for use of alternative treatment strategies. Probiotics have been reported to have the ability to interfere with enteric pathogens and play a role in inducing interruptions of the earlier interactions of the pathogens to the host cells [50]. Therefore the use of probiotics in pathogen inhibitions is favoured more than that of antibiotics. In order to assess how adaptation to stress factors affect the inhibitory activity of the probiotics against pathogens, the inhibitory effect of the stress adapted single probiotics strains was compared to that of cocktails comprising cells of different stress- adapted strains and one containing all non- adapted cells.

**Figure 3:**
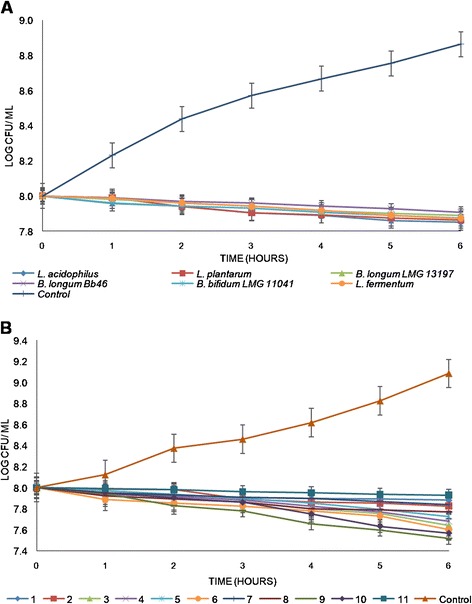
**The antagonistic effects of (A) single {multi-stress adapted} and (B) combination {non-adapted; multi-stress-adapted} probiotics on growth of** ***S*** **.** ***aureus*** **over a period of six hours.** *Each point represents the mean of three independent experiments, error bars are standard deviations.*

The inhibitory effect of multi-stress adapted single strain probiotic against *S. aureus* is shown in Figure 3A. The numbers of *S. aureus* incubated in the absence of probiotics increased throughout the 6 h of incubation from initial count of 8 log cfu/ml to 8.864 log cfu/ml, an increase close to 1 log (0.9). However, when inoculated together with probiotics, the numbers of *S. aureus* decreased in the presence of all strains. *L. acidophilus* La14 150B reduced the counts of *S. aureus* from 8.00 to 7.850 log cfu/ ml, it was the culture that inhibited *S. aureus* better than the other single cultures. *L. acidophilus* La14 150B had 0.15 log CFU/ ml difference compared to 0.136, 0.127, 0.124, 0.109 and 0.092 log cfu/ ml for *L. plantarum*, *L. fermentum*, *B. bifidum* LMG 11041, *B. longum* LMG 13197 and B. *longum* Bb 46, respectively from the highest inhibition to the lowest. There was a significant difference in numbers of *S. aureus* in the presence and absence of probiotic, but there was no significant difference between the different probiotic strains. When comparing inhibitory effects of combinations of probiotics it was interesting to observe that a cocktail containing all the six non-adapted probiotic strains was the least effective in inhibiting growth of *S. aureus*, reducing counts by only 0.07 log cfu/g (Figure 3B). Combination 9 was the cocktail of multi-stress adapted probiotics which best inhibited *S. aureus*, whereby it reduced *S. aureus* counts from 8.00 to 7.519 log cfu/ml, a difference of 0. 481 compared to 0.119, 0.174, 0.357, 0.319, 0.276, 0.398, 0.161, 0.229, 0.432 log cfu/ml for combinations 1, 2, 3, 4, 5, 6, 7, 8 and 10 respectively. Similar to what was observed for single probiotics, growth of *S. aureus* in the absence of probiotics increased by 1.08 log cfu/ml during incubation period.

### Antagonistic effects of single and probiotic cocktails on *E. coli*

**Figure 4:**
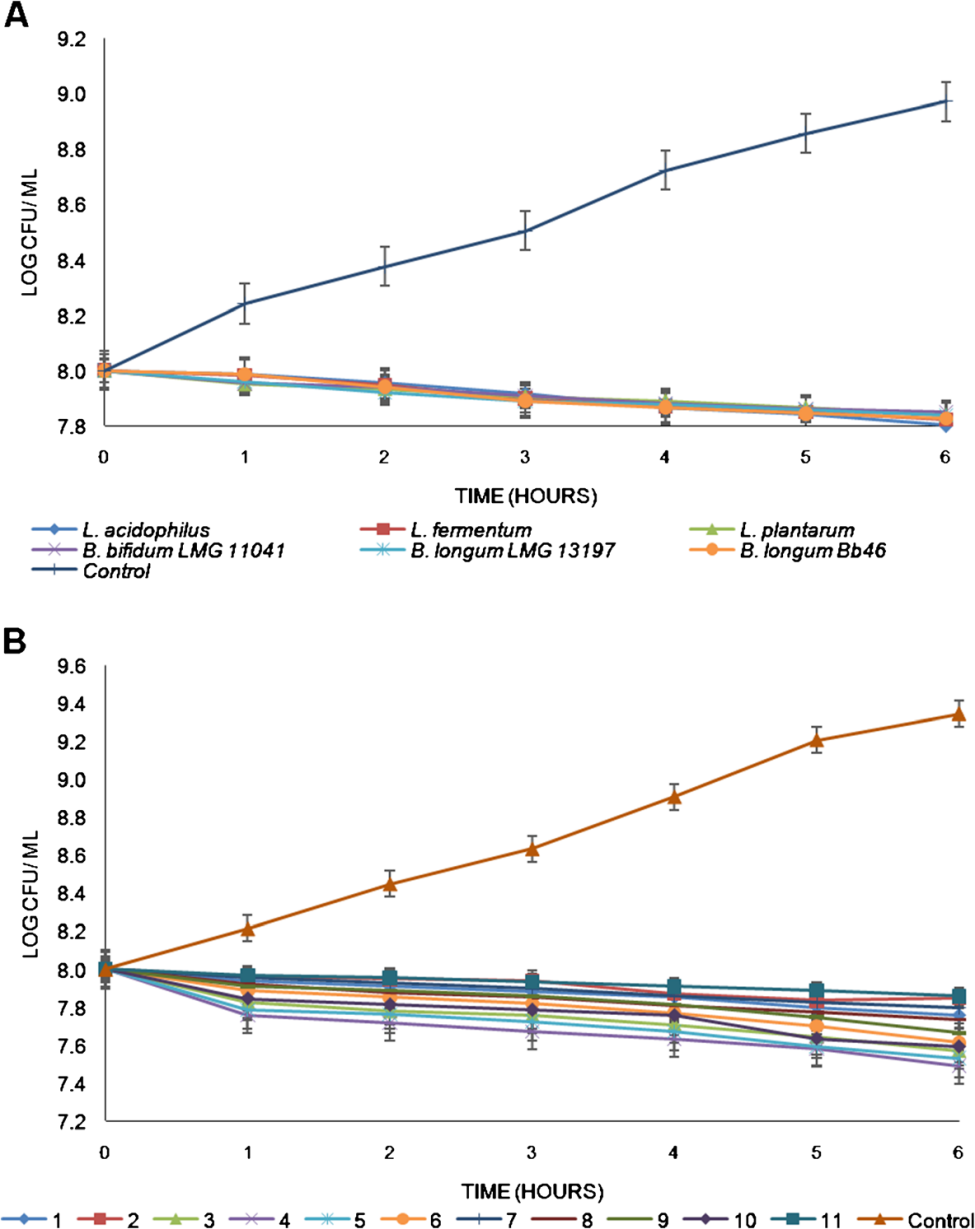
**The antagonistic effects of (A) single {multi-stress adapted} and (B) combination {non-adapted; multi-stress-adapted} probiotics on growth of** ***E*** **.** ***coli*** **over a period of six hours.** *Each point represents the mean of three independent* experiments, *error bars are standard deviations*.

We also investigated the inhibition of *Escherichia coli* by singular and cocktails of multi-stress adapted probiotic strains (Figure 4). Similar to what was observed for *S. aureus*, *L. acidophilus* La14 150B was the most effective in inhibiting pathogen growth, showing a reduction in viable *E. coli* counts by 0.198 log cfu/ ml compared to 0.178, 0.174, 0.161, 0.160 and 0.150 log cfu/ ml for *L. plantarum*, *L. fermentum*, *B. bifidum* LMG 11041, *B. longum* LMG 13197 and *B. longum* Bb 46, respectively (Figure 4A). The control culture increased throughout incubation time by 0.971 log cfu/ ml. Combination 4 reduced *E. coli* better than the other combinations from 8.00 to 7.491 log cfu/ ml difference of 0.509 log cfu/ ml compared to 0.244, 0.151, 0.432, 0.469, 0.387, 0.201, 0.266, 0.337 and 0.409 log cfu/ ml for combinations 1, 2, 3, 5, 6, 7, 8, 9 and 10, respectively (Figure 4B). As was observed for *S. aureus*, a cocktail of all the six non-adapted probiotic strains was the least effective in controlling growth of *E. coli*, resulting in 0.143 log cfu/ml reductions in numbers of viable *E. coli* during the six hours of incubation. Viable numbers of *E. coli* incubated in absence of probiotics increased by 1.344 log cfu/ ml (Figure 4B).

When we used the single probiotic cultures to inhibit the pathogens, *S. aureus* and *E. coli*, we reported the same order of inhibition in both *S. aureus* and *E. coli*. All the *Lactobacilli* strains were more aggressive and had better inhibitory effects against the tested pathogens than *Bifidobacteria*, indicating that they maintained their inhibitory effects. Although multi-stress adaptation improve inhibitory effects of *Bifidobacteria*, in terms of performance they could still not outperform the *Lactobacilli*. We could therefore conclude that the single *Lactobacilli* cultures are more aggressive and have better inhibitory effects than the *Bifidobacteria* culture. Superior inhibitory effects of *Lactobacilli* than *Bifidobacteria* have been reported elsewhere [51-53], therefore this trend was the same even after pre-adaptation to multiple stresses. The antagonistic effects of the probiotic cells towards the pathogens are mostly related to the ability of the strain to excrete the broad spectrum antimicrobial substances [54]. Therefore, the results suggest that exposure of the probiotics did not have negative effects on the ability of the probiotics to excrete the antimicrobial substances, a phenotype that is directly linked to pathogen inhibitory abilities of probiotics.

When we studied the inhibitions of the different stress- adapted combinations compared to the combination of the non- adapted cells, we wanted to look at ways to enhance the inhibition of the pathogens and also as to whether the use of stress adapted cells in combinations will have an effect on the inhibitions. In our results we report that cocktails of multi- stress adapted probiotics strains had better pathogen inhibition effects than a cocktail of non- adapted combination. Inhibition of *S. aureus* by combination 9, a cocktail of multi-stress adapted probiotics which best inhibited growth of this pathogen, was significantly better than its inhibition by a cocktail of the six non-adapted cells (p = 0.01). Similarly, there was a significant difference between inhibition of *E. coli* by combination 4, a cocktail of multi-stress adapted cells that best inhibited *E. coli*, and its inhibition by a cocktail of all the six non-adapted cells (p = 0.003). This indicates that pre- adaptation of probiotics to multiple stresses enhanced their antipathogenic effects. The main advantage of using probiotic mixtures is that they have beneficial effects against a wide range of disorders [55]. This suggests that use of probiotic mixtures can be very important in many clinical models. Collado et al. [56], used the single and combination probiotics to inhibit pathogens from adhering to the human intestinal mucus. In their results they found that all the single probiotics inhibited the pathogens and that not only did their combination probiotics inhibit the pathogens, they enhanced the inhibition percentages than when the single strains were used. In our study the same results were found, only we used the single stress- adapted and their combination probiotics. We hypothesized that the use of the stress- adapted combinations will have a better effect than the single stress adapted and a combination of the non- adapted cells, and we therefore accept this hypothesis. The enhancement of the pathogen inhibitions will therefore be useful in the probiotic concept. We showed that the use of pre- adapted combination probiotics enhances the inhibition of the pathogens. Therefore, the combination of using enhanced probiotic strains, in this case the stress adapted probiotic combination with different strategies such as the pre- incubation of the intestinal epithelial cells would therefore results in further inhibition of the pathogens.

## Conclusion

Firstly, the adapted cells performed better in the GIT conditions than the non- adapted cells showing that the multi stress pre- adaptation is a safe mechanism for enhancing the viability of probiotics under unfavourable conditions. Secondly, the combination of the adapted cultures has better inhibitory effects than the adapted single strain cultures and the combination of the non- adapted cultures and the single cultures on pathogenic *E. coli* and *S. aureus.*

## Materials and methods

### Bacterial cultures

*Bifidobacterium bifidum* LMG 11041, *Bifidobacterium longum* LMG 13197, *Bifidobacterium longum* Bb46, *Lactobacillus acidophilus* La14 150B, *Lactobacillus fermentum* and *Lactobacillus plantarum* glycerol stock cultures from our laboratory were used as test probiotic cultures while *Escherichia coli* and *Staphylococcus aureus* were used as select foodborne pathogens for the antipathogenic tests. *Lactobacillus* spp. were sub-cultured in de Man Rogosa and Sharpe (MRS) broth (Merck, South Africa) and *Bifidobacterium* spp. in MRS (supplemented with 0.05% v/v L- cysteine hydrochloride monohydrate) (MRS- cys- HCl), followed by incubation at 37°C for 72 h in anaerobic jars containing Anaerocult A gaspacks. After the final subculturing, the initial concentration of probiotic bacteria present was determined by serially diluting the cultures in ¼ strength Ringer’s solution, followed by pour plating onto MRS and MRS-cys- HCl plates in triplicates, for *Lactobacillus* and *Bifidobacterium* spp., respectively. The plates were incubated anaerobically at 37° C for 72 hours. The adapted and the non- adapted cells were normalised to an optical density of 0.2 at 600 nm which is approximately equivalent to 10^8^ cfu/ ml in the different experiments.

*Escherichia coli* and *Staphylococcus aureus* were cultured in Luria Bertani (LB) broth, incubated in an Orbital shaker incubator LM- 530R, 100 rpm at 37°C. The concentration were determined by plating of subcultures on Mannitol salt agar (Merck, SA) and MacConkey agar (Merck, SA) plates for *S. aureus* and *E. coli*, respectively. The plates were then incubated at 37°C for 48 hours. All the cultures were subcultured twice before their use in experiments.

### Stress adaptation of probiotics

#### Acid adaptation

Overnight broth cultures of the probiotics were harvested by centrifugation at 3000 rpm for 15 min using a Mini spin Eppendorf centrifuge. The pellets were resuspended in 1 ml of ¼ strength Ringer’s solution (Merck, South Africa). Then 1 ml of these cultures were added to separate tubes containing 9 ml MRS broth adjusted to pH 2 using 1 M HCl. The cultures were then incubated at 37°C and 100 μl subsample at 120 minutes were transferred to 900 μl MRS or MRS- cys- HCl broth. The suspensions were then serially diluted up to 10^−7^ using ¼ strength Ringer’s solution and 0.1 ml of each dilution was pour plated onto MRS or MRS-cys- HCl plates in triplicates. The plates were incubated anaerobically at 37° C for 72 hours. The colonies of the plates containing 30–300 colonies were counted. The cultures (pre- exposed to acid) were recovered by growing them overnight in MRS or MRS- cys- HCl broth. They were taken as the acid adapted strains and were subsequently used for the bile adaptation process.

### Bile adaptation

Ten millilitres of the overnight cultures of the acid adapted strains were aseptically transferred into Falcon tubes containing 2.0% (w/v) bile solution (pre- weighed). The flasks were then incubated anaerobically in a shaking incubator (100 rpm) at 37°C. At 60 minutes, 1 ml aliquots were harvested and added to 9 ml MRS-cys- HCl broth. The suspensions were then serially diluted up to 10^−7^ using ¼ strength Ringer’s solution and 0.1 ml of each dilution was pour plated onto MRS or MRS-cys- HCl plates in triplicates. The plates were incubated anaerobically at 37° C for 72 hours. The colonies of the plates containing 30–300 colonies were counted. The surviving cells were recovered by growing them on MRS or MRS- cys- HCl agar plates incubated anaerobically in anaerobic jars with Anaerocult A gaspacks and Anaerotest strips for 72 hours. The cells that survived after the exposure to 2.0% bile for 60 minutes were used further for the temperature adaptation.

### Temperature adaptation

Overnight cultures of acid- bile adapted strains grown in MRS or MRS- cys- HCl broth at 37°C in a shaking incubator at 100 rpm were used. One millilitre of the overnight culture was added to nine millilitres of fresh MRS/ MRS- cys- HCl broth and the cultures were incubated at 55°C (AccuBlock digital dry bath). Hundred microliters were withdrawn after 120 minutes and added to 900 μl MRS or MRS- cys- HCl broth. The suspensions were then serially diluted up to 10^−7^ using ¼ strength Ringer’s solution and 0.1 ml of each dilution was pour plated onto MRS or MRS-cys- HCl plates in triplicates. The plates were incubated anaerobically at 37° C for 72 hours. The colonies of the plates containing 30–300 colonies were counted. The cultures were recovered by growing them overnight in MRS or MRC- cys- HCl broth at 37°C. These acid- bile- temperature adapted strains were stored in 20% glycerol (1:1) at −20°C.

### Viable plate count

The non- adapted and the adapted cells were grown overnight in MRS or MRS- cys- HCl broth at 37°C were used. The cells were suspended in ¼ strength Ringer’s solution. The suspensions were then serially diluted up to 10^−7^ using ¼ strength Ringer’s solution and0.1 ml of each dilution was pour plated onto MRS or MRS-cys- HCl plates in triplicates. The plates were incubated anaerobically at 37° C for 72 hours. The colonies of the plates containing 30–300 colonies were counted and this gave the initial amount of bacteria present before the cells were exposed to stress adaptation.

### Survival under the git conditions

#### Acid tolerance

The investigation of the tolerance of the non- adapted and the stress adapted cells to acid was done using method that was described by Brashears et al. [57], with minor modifications. Briefly cultures of the non- adapted and adapted cells of lactobacilli spp. and bifidobacterial spp. were grown in MRS or MRS- cys- HCl at 37°C overnight in a shaking incubator at 100 rpm. The cultures were sub-cultured into 10 ml of fresh MRS or MRS- cys- HCl broth adjusted to different pH values (2, 2.5 and 3) with 1 M HCl followed by incubation at 37°C in a shaking incubator (100 rpm). Then 100 μl aliquots were harvested at 60, 120 and 180 minutes, transferred into 10 ml MRS/ MRS- cys- HCl broth. The suspensions were then serially diluted up to 10^−7^ using ¼ strength Ringer’s solution and 0.1 ml of each dilution was pour plated onto MRS or MRS-cys- HCl plates in triplicates. The plates were incubated anaerobically in anaerobic jars with Anaerocult A gaspacks and Anaerotest strips at 37° C for 72 hours. The colonies of the plates containing 30–300 colonies were counted.

### Tolerance to bile salts

Tolerance of the probiotic cultures to bile was performed using a method by Tsai et al. [58] with minor modifications. Briefly, overnight broth cultures of both the adapted and the non- adapted lactobacilli spp. and bifidobacterial spp*.* were harvested by centrifugation at 3000 rpm for ten minutes. The pellets were washed in ¼ strength Ringer’s solution and mixed by vortexing for 30 seconds. Then 100 μl of the solution was added to MRS or MRS- cys- HCl broth adjusted to 1, 2 and 3% (w/v) bile concentration and grown in a shaking incubator at 37°C with the readings taken every hour for 3 hours. Cultures inoculated in 0% bile were used as controls. The suspensions were then serially diluted up to 10^−7^ using ¼ strength Ringer’s solution and 0.1 ml of each dilution was pour plated onto MRS or MRS-cys- HCl plates in triplicates. The plates were incubated anaerobically in anaerobic jars with Anaerocult A gaspacks and Anaerotest strips at 37° C for 72 hours. The colonies of the plates containing 30–300 colonies were counted.

### Preparation of simulated gastric and intestinal fluids

The simulated gastric juices were prepared by briefly suspending 3 g/ l of pepsin (Merck, SA) in saline (0.5% w/v) and adjusted to 2.0 with 1 M HCl. The simulated intestinal fluid was prepared by dissolving 6.8 g monobasic potassium phosphate (Merck, SA) into 250 ml distilled water. 77 ml of NaOH (0.2 M) was added and mixed. 500 ml of distilled water was then added and the solution was mixed by vortexing for 30 s. Then 10 g of pancreatin was added and mixed and the solution was adjusted to pH 6.8 with 1 M NaOH or 1 M HCl. The solution was then made up to 1000 ml.

### Exposure to gastric and intestinal conditions

The non- adapted and adapted cultures of *Lactobacilli* spp. and *Bifidobacteria* spp. grown overnight in MRS broth and MRS- cys- HCl broth, respectively. Aliquots of 1 ml were added to 9 ml of simulated gastric fluid (pH 2) for 2 h at 37°C. After 2 h, 0.1 ml of the solution was withdrew and added into 0.9 ml of the simulated intestinal fluid (pH 6.8) for 2 h at 37°C. Then 100 microliters was withdrawn from the tubes and plated in triplicates onto MRS or MRS- cys- HCl agar plates. The plates were incubated anaerobically in anaerobic jars with Anaerocult A gaspacks and Anaerotest strips at 37°C for 72 hours. The colonies of the plates containing 30–300 colonies were counted.

### Preparation of probiotic combinations

The six acid- bile- temperature adapted and the six non-adapted *Lactobacilli* and *Bifidobacteria* cultures were used for the preparation of combinations. They were grown overnight in MRS or MRS- cys- HCl broth. The probiotic cultures suspensions were prepared for each culture to achieve an optical density of 0.2 at 600 nm (OD_600_) were used. They were then added in equal amounts to make different combinations. There were 54 different combinations from the six cultures. From the 54 combinations, we then tested for their acid tolerance, the bile tolerance and the subsequent exposure to the gastric and the intestinal conditions (Data not shown). From there we chose the 10 best tolerant combinations and one combination of the six non- adapted cells (Table 3). The probiotic combination cultures were then stored in a ratio of 1:1 bacterial culture: 20% glycerol stock at 20°C.

**Table 3:** **The different probiotic combinations prepared by adding equal concentrations (10** ^**8**^ **cfu/ ml) of probiotic strains**

**Combination number**	**Probiotic strains**
1	*L. acidophilus* La14 150B *+ L. plantarum + B. longum* LMG 13197
2	*L. acidophilus* La14 150B *+ L. plantarum + B. bifidum* LMG 11041
3	*L. acidophilus* La14 150B *+ L. fermentum + B. longum* Bb46
4	*L. acidophilus* La14 150B *+ B. longum* Bb46 *+ B. bifidum* LMG 11041
5	*L. acidophilus* La14 150B *+ B. longum* LMG 13197 *+ B. bifidum* LMG 11041
6	*L. plantarum + L. fermentum + B. bifidum* LMG 11041
7	*L. plantarum + L. fermentum + B. longum* LMG 13197
8	*L. plantarum + L. fermentum + B. longum* Bb46
9	*L. plantarum + B. longum* Bb46 *+ B. longum* LMG 13197
10	*L. acidophilus* La14 150B *+ L. plantarum + L. fermentum + B. longum* LMG 13197 *+ B. longum* Bb46 *+ B. bifidum* LMG 11041 (All adapted)
11	*L. acidophilus* La14 150B *+ L. plantarum + L. fermentum + B. longum* LMG 13197 *+ B. longum* Bb46 *+ B. bifidum* LMG 11041 (All non- Adapted)

The combinations were prepared by adding the cells in a ratio of 1:1. The different combinations used here are indicated in the table with their corresponding combination numbers.

### Antagonistic tests

The pathogenic cultures of *E. coli* and *S. aureus* were used for this experiment. They were grown in LB broth overnight at 37°C. Bacterial suspensions were prepared in sterile water for each of the pathogens to achieve an optical density of 0.2 at 600 nm which corresponds to approximately 1 × 10^8^ cfu/ ml. The method that was used for the antagonistic tests was adapted from Jamalifar et al. [32] with minor modifications. Briefly, 15 ml of 1 × 10^8^ cfu/ml probiotic combination cultures were added into flasks containing 100 ml LB broth and to that 1 ml of 1 × 10^8^ cfu/ml of the pathogen was added. The control flasks did not contain any probiotics. The flasks were incubated in a shaking incubator (100 rpm) at 37°C for 6 hours. Hundred microliter subsamples were withdrawn from the flasks hourly, diluted in 900 μl of ¼ strength Ringers solution, then 100 μl were plated in triplicates onto Mannitol salt agar (Merck, SA) and MacConkey agar (Merck, SA) plates for *S. aureus* and *E. coli*, respectively. The plates were then incubated at 37°C for 24 hours.

### Statistical analysis

Statistical analysis of the difference between the adapted and the non- adapted strains was analysed by using the two- way Student *t- test* from the software Statistica v10. Where a P- values < 0.05 was considered to be statistically significant and P- values > 0.05, statistically non- significant.

## Abbreviations

GIT: Gastrointestinal Tract
cfu/ ml: Colony forming unit per millilitre
OD: Optical density
MRS: de Man, Rogosa and Sharpe
LB: Luria Bertani
rpm: Revolutions per minute
nm: Nanometre
